# Parents’ Views on Autopsy, Organ Donation, and Research Donation After Neonatal Death

**DOI:** 10.1001/jamanetworkopen.2023.41533

**Published:** 2023-11-06

**Authors:** Elizabeth E. Crouch, Carlos Damas, William C. Bartrug, Anne Shamiyeh, Meghan Scelfo, Madeleine Dreyfus, Dawn Gano, Summer Segal, Linda S. Franck

**Affiliations:** 1 Department of Pediatrics, University of California San Francisco; 2 The Eli and Edythe Broad Center of Regeneration Medicine and Stem Cell Research, University of California San Francisco; 3 Helping After Neonatal Death of the Bay Area, Redwood City, California; 4 Division of Pediatric Neurology, Department of Neurology, Benioff Children’s Hospital, University of California San Francisco; 5 Department of Pediatrics, Stad Center for Pain Palliative and Integrative Medicine, University of California San Francisco; 6 School of Nursing, University of California San Francisco

## Abstract

**Importance:**

Parents who experience neonatal loss have the option to participate in autopsy, organ donation, and research donation. However, clinicians are uncomfortable discussing autopsy and may not be aware of research and organ donation opportunities.

**Objective:**

To capture the perspectives of parents who had experienced neonatal loss about autopsy, organ donation, and research donation.

**Design, Setting, and Participants:**

This qualitative study used virtual focus groups with parents who attended a local bereavement support group in the US. Participants were recruited from Helping After Neonatal Death, a support group with a local chapter. Participants self-selected from an email request if they met the following criteria: aged 18 years or older, English speaking, at least 6 months elapsed since neonatal death, and access to a video conference device with internet. Focus groups took place between April and September 2021. The recorded sessions were analyzed using a grounded theory–informed approach by the research team that included parents with experience of neonatal loss. Data were analyzed from December 2021 through December 2022.

**Results:**

A total of 14 mothers engaged in the focus group; 9 (75%) were aged 30 to 39 years, and 8 (66%) were White. The mothers were overall well educated. The first main theme grew from the lived experience of neonatal loss, specifically the importance of offering all parents the option to donate, rather than prejudging who would or would not be interested. Parents of neonates who die have few opportunities to parent that child and make loving decisions for them. Participants emphasized that the conversation about autopsy, organ donation, and research donation, albeit difficult, can offer a meaningful parenting experience. A second main theme that emerged related to how organ or tissue donation could provide additional meaning to a child’s life. These choices contributed to building a legacy to honor their child’s memory, which also helped with grief and coping with their loss. A third theme included recommendations to clinicians and health systems for improving communication, including written information for parents and communication training for health care professionals.

**Conclusions and Relevance:**

In this qualitative study, parents who experienced neonatal loss endorsed the importance of offering parents the choice of autopsy, organ donation, or research donation with skillful and empathetic communication. They provided practical recommendations to improve communication and empower families.

## Key Points

**Question:**

Do parents who experience neonatal loss want to engage in discussions around autopsy, organ donation, and research donation?

**Findings:**

In this qualitative research study, 14 bereaved parents communicated their desire to know the options for contributing to education and research after the death of their infant. Participating in these options can contribute to meaning-making, a central component of grief processing, and engaging in this decision provides an opportunity to parent their child even for families who decline.

**Meaning:**

These findings suggest that although conversations about autopsy, organ donation, or research donation are challenging, parents want to be informed and make their own choices.

## Teaser

This qualitative study evaluates parents’ view on autopsy, organ donation, and research donation after neonatal death.

## Introduction

In certain high-risk populations, such as extremely low birth weight infants, mortality rates are up to 40%, even in highly specialized centers in the US.^1^ Autopsy and organ, tissue, or cellular donation for transplantation or research may be offered to families in this situation. Despite concerns about causing further distress for bereaved parents, these options can contribute to meaning-making, a central component of grief processing.^2,3,4,5^ Among potential benefits for families, up to 48% of neonatal autopsies show findings that were previously unknown according to clinical and radiographic tests.^6^ Autopsy in the context of neonatal death facilitated future family planning, addressed the family’s altruistic desire to prevent future neonatal death, helped to explain their loss, and aided in the grief process by fostering a sense of emotional closure.^7,8^ Furthermore, autopsy can add to results from genetic testing and diagnose a hereditary condition that may impact family members.^9^ Due to public health campaigns, the benefits of organ donation are more readily apparent to pediatric and neonatal families.^10^ Although neonatal patients are rarely able to provide donated organs due to their size, engaging the family to ask their preference surrounding organ donation can be perceived as validating the child’s life whether or not the organs are suitable for donation.^10,11^

Despite the significant potential benefits to families of autopsy, organ donation, and research donation, clinicians rarely receive formal training to skillfully conduct these discussions.^12,13,14^ Hurried, insensitive conversations about autopsy contribute to a family’s emotional distress.^7^ With insufficient training, clinicians are appropriately concerned about retraumatizing families and some completely avoid the topic.^15,16,17^ Although few pediatric and neonatal families are offered organ and research donation,^10^ a growing online presence has improved networking and support for families with children who have rare and serious conditions. This community has increased the numbers of parents interested in research donation.^5,18^

Guiding families through neonatal loss should be individualized.^19^ Despite the high stakes of this conversation, few neonatal intensive care units (NICU) have a standard process for discussions with families about autopsy, organ donation, or research donation, leading to uneven access, racial and cultural inequities in who is approached, and potential regret.^18^ Critically, the voices of families who experience neonatal loss are underrepresented in the literature. The objective of our study was to engage families who experienced neonatal death and ask about their participation and interest in autopsy, organ donation, and research donation. In addition, we invited their ideas for best practices in communication about these important and sensitive topics.

## Methods

### Design

We formed a steering committee to design the study, including a neonatologist (E.E.C.), a pediatric resident (C.D.), 3 parents who had experienced neonatal loss (A.S., M.S., and W.C.B.), an NICU nurse (W.C.B.), a support group facilitator (A.S.), an NICU social worker (M.D.), a neonatal neurologist (D.G.), a counselor who specializes in pediatric and perinatal palliative care (S.S.), and a nurse researcher in family-centered care (L.S.F.). We then formed a partnership with Helping After Neonatal Death (HAND),^20^ a local support group in the US for parents who have experienced neonatal loss. This study protocol was approved by the University of California, San Francisco institutional review board. Participants provided oral informed consent and were emailed the facilitator’s guide with topics and questions for discussions before the focus groups. We followed the Consolidated Criteria for Reporting Qualitative Research (COREQ) reporting guidelines.

### Recruitment

We recruited participants by sending a flyer via email to the listserv of local HAND support groups. Inclusion criteria were being aged 18 years or older, speaking English, at least 6 months elapsed since neonatal death, and having access to a video conference device with internet. Participants were compensated $50 for each focus group session or $100 total after completing 2 sessions. The focus groups took place between April and September 2021.

### Data Collection

Each semistructured session was conducted in English and cofacilitated by a researcher (E.E.C. and C.D.) and parent bereavement specialist (A.S.) from HAND. Our steering committee designed the facilitator guide (eAppendix in Supplement 1), which explored how families were informed about and made decisions after neonatal loss. The sessions were recorded and transcribed verbatim. Self-reported demographic data including age group and race and ethnicity were collected via a brief emailed survey. Race and ethnicity were collected to understand the population of our study and what groups may be underrepresented: different groups may bring unique experiences that are informed by their various identities.

### Data Analysis

To analyze the data, we selected a grounded theory–informed approach because of its open-ended philosophy for a fairly unexplored topic.^11,21,22^ Its selection was also strengthened by the prevalence of grounded theory in the health care setting and its previous use in autopsy and organ donation research specifically.^21,22^ In line with a grounded theory approach, we analyzed the data without preconceived theoretical ideas or hypotheses, instead allowing concepts and themes to emerge during the iterative process of data collection and analysis.^11,21^ All transcripts were coded using Dedoose software version 9.0.107 (Dedoose) by at least 2 authors, including W.J.B., E.E.C., and C.D., representing expertise in neonatology, general pediatrics, nursing, health disparities, family advocacy, and qualitative analysis. The steering committee met at regular intervals to review and revise the coding structure. Following coding, the group met to develop themes from the codes. Data collection ended when thematic saturation was reached. In addition to using multiple coders, reflexive discussions, documented procedures, and audit trails were used to increase trustworthiness and credibility of the findings. In addition, the themes and subthemes were shared with participants and feedback sought.^23^ Data were analyzed from December 2021 through December 2022.

## Results

**Figure 1.**
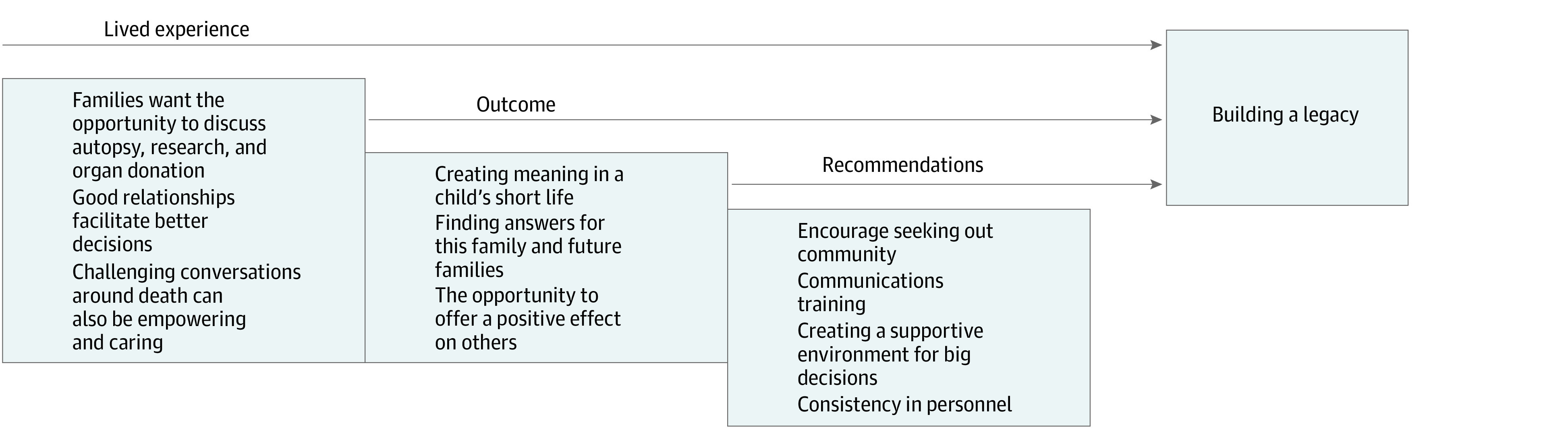
The Major Themes and Subthemes The major and subthemes of this study, which all contribute to building a legacy to honor children who have died.

Three virtual focus groups were conducted, with two 90-minute sessions for each group. A total of 27 families initially expressed interest in participation. We then arranged focus groups at times that best fit the participants’ availability. The final sample consisted of 2 groups of 5 parents and 1 group with 4 parents, representing 14 different families. All participants identified as mothers; 9 (75%) were aged 30 to 39 years, and 8 (66%) were White. Participant demographics are shown in Table 1. From 8 main codes and 17 subcodes, 3 themes emerged from our focus groups: (1) the parents’ lived experiences around autopsy, organ donation, and research donation in the context of neonatal loss; (2) how these options can contribute to building a legacy to honor their child’s life; and (3) heath system recommendations for providing a supportive environment around these decisions. An overview of these themes and subthemes are described in Figure 1, and exemplar quotes are provided in Table 2.

**Table 1.** Participant Characteristics

Characteristic	**Participants, No. (%)** ^a^
Sex	
Women (mothers)	12 (100)
Age, y	
30-39	9 (25)
40-49	3 (7)
Marital status	
Married	12 (100)
Do you have other living children?	
Yes	10 (83)
No	2 (17)
Educational level	
College degree	4 (33)
Graduate-level degree	8 (66)
Insurance type	
Private	12 (100)
Racial and ethnic background	
Asian	1 (8)
Indian	1 (8)
Mixed race	2 (16)
White	8 (66)

^a^ A total of 14 participants were interviewed but 2 declined to provide demographic information.

**Table 2.** Major Themes and Subthemes and Representative Quotes

Major themes and subthemes	Exemplar quotes
Lived experience	
Discussing autopsy, organ donation, and research donation can be challenging, empowering, and caring	“It was clear to me that the doctors also wanted the autopsy. And for me, that actually felt nice. For me, it felt like the doctors were really invested in wanting to know what happened because it was this total, out of nowhere thing. And that gave me a sense of comfort that they were invested in knowing as well.” (Participant 1.4)
Not being offered leaves questions	“Nobody asked me if I wanted to donate his body through research or if I wanted to donate his organs. It was not an option that was given to me, it’s an option I would have taken had it been offered to me.” (Participant 1.2)
Details matter: when, by whom, and which parents are involved	“But there was also other things like the genetic testing and stuff that they never asked me about. They asked my husband, and he turned it down. I was really upset to hear about that years later; I did not know that they avoided the mother. I don’t know if it was like...‘I didn’t want to emotionally upset her.’ I was upset because I really still wanted to know.” (Participant 3.1)
Building a legacy	
Making meaning of child’s short life	“I strongly believe that if the circumstance permits, every single doctor should inquire whether a patient, the baby, is a candidate for organ donation with their expertise because as you know, just in and we’ve all said that it just adds another level of meaning and layer to our children’s lives that are cut short.” (Participant 2.4)
Finding answers	“It was a really quick conversation, mostly because my husband and I both just had a really intuitive certain reaction of, ‘Yes, absolutely.’ I think for both of us, we at the time had an unspoken understanding, and now we have a spoken understanding of just for us, it was healing to be able to help any other parents in our situation not have to get to the point that we were at.” (Participant 1.4)
A positive impact for others	“[The physician] came back and told us...that there would be 5 researchers that would be receiving her samples, that they would take 11 samples including her lungs and her heart and her brain to study autism brain development and other neurological disorders and...all the stuff that her life would contribute to...it put it in real terms of, okay, this is meaningful. This is what we want to do and so, we were fully on board with it.” (Participant 2.4)
Parent recommendations	
Partnership and collective experience	“I would say something to the extent of, after you’ve apologized for the loss and comforted them, say, when I do bereavement training...we talk about using the collective experience: ‘So many families have found it comforting to seek out answers for what may have caused the death.’” (Participant 2.4)
Communications training and expertise	“I have really positive memories of what nurses said to me and I have really negative memories of what nurses said to me. And everything in that moment of trauma is getting deeply ingrained into your brain. Unfortunately that’s just how it’s working. So if everybody is trained on how to deal with it, I think like the more we can support, and the more we can ask from patients too in that space if they feel supported and safe.” (Participant 3.1)
Supportive environment during decision-making and with bereavement	“[Y]ou always say the baby’s name. Ask, ‘Did you get a chance to name your child? What is your child’s name?’ And then, from that point on, always refer to the child by their name. And then you can say, ‘[The baby’s] donations will help this research, and she will be a valuable member of the research team,’ so making her be almost like a colleague, almost part of the valuable work that is being done.” (Participant 2.4)
Preparation whenever possible	“I like written materials, to look at and then, give me a chance to think about it before I have the conversation, talk to my husband about it, just let it sit for a little bit before we talk about it.” (Participant 2.5)
Consistency in personnel	“So when you have multiple people, it can create the experience of it being very dehumanizing, I feel, for the parents involved. So I personally, would be an advocate for the doctor that has been the primary caregiver for that patient, being there to talk about it, in conjunction with the research coordinator, so there’s a friendly face with the new face, who has the standardized, appropriate, emotional language to discuss. That would be the ideal situation.” (Participant 2.4)

### Lived Experience

In the face of neonatal loss, our participants strongly supported discussing autopsy, organ donation, and research donation with all affected families when feasible. They reported that these conversations can be challenging, but also provided benefits in terms of the parent’s ability to process the loss and feel supported by the medical team. Participant 1.4 reported, “We were offered autopsy and we did do it. It was really helpful, especially because Coco’s passing was such a surprise to us...the autopsy did actually help us get some good closure on what happened.” Furthermore, Participant 1.4 said, “It was clear to me that the doctors also wanted the autopsy....For me, it felt like the doctors were really invested in wanting to know what happened because it was this total, out of nowhere thing. And that gave me a sense of comfort.” Some of the challenges relayed were the timing of the discussion, the comfort and knowledge of the medical professionals leading the conversation, and factors related to the family. Participant 1.4 also described a frustrating situation where the clinicians were not aware that autopsy was a free service: “Another person would come in later and be like, ‘Oh, do you want to do an autopsy?’ And I’m like, ‘Yes, I’ve asked 2 or 3 times how much does it cost? And then we’ll make our fully informed decision.’” The participants offered differing perspectives on their ability to engage in these discussions before or after the death. For example, Participant 3.3 said, “I do envision it (the conversation) beforehand...once Theo passed, I wanted to leave the hospital as fast as possible...I just wanted to be home and back into my own comfort.” In contrast, some parents wanted to focus on the remaining moments with their child as described by Participant 3.2, “We had just 2 hours with her....I couldn’t imagine in that 2 hours having to take time away (to discuss autopsy, organ donation, and research donation).” Participant 3.3’s experience offers one solution; she said, “I remember when they came to speak with us about the arrangements, they stopped and asked if it was an okay time to talk about that. And I felt like that was...helpful.”

When framed with compassion and support, these options can empower families and provide transparency about the baby’s medical course. Parents of neonates who died have few opportunities to parent that child and make loving decisions for them. The conversation about autopsy, organ donation, and research donation can offer a meaningful parenting experience under appropriate conditions. The autopsy results can be helpful whether they identify a cause of death or not; parents reported that negative findings could offer reassurance that there was nothing else they could have done and provide closure. As Participant 2.4 said, “I know it’s, to each their own, but for me, the more answers the better.” On the other hand, not fully discussing parental options can lead to regret when parents realize what was possible at a future date. Participant 1.2 said, “But I am an organ donor. The father is alive today because he received an organ donation. I have strong feelings about it and would have wanted to do that (organ donation), and simply wasn’t asked.” These experiences point to the critical importance of the clinicians’ knowledge on the educational value of autopsy and whether organ and/or research donation is possible before informing the family. These pieces will allow a thoughtful discussion on the family’s wishes after death.

### Building a Legacy

Even during such a challenging time, parents reported receiving comfort at the idea of their child contributing to society through their loss. Participant 2.4 describes, “I strongly believe that if the circumstance permits, every single doctor should inquire whether a patient, the baby, is a candidate for organ donation because...it just adds another level of meaning and layer to our children’s lives that are cut short.” Most poignantly, parents reported that one of the greatest tragedies of losing a child so early in life was the missed opportunity to parent the child and enjoy their presence. Another parent reported that it was difficult to speak about her baby in the presence of other families. They often did not know how to respond to her, and this isolation brought additional sadness. Engaging in organ or research donation offered families a different way to talk about their experience and provided opportunities to remember and talk about their loved child.

### Parent Recommendations

Parents who experienced neonatal loss had many recommendations for a standardized approach in these challenging situations. One key idea centered around relationships that support parents at this time and lessen isolation. Because of the high-risk NICU population, these conversations often take place in the middle of the night or on weekends. Therefore, all personnel who work in neonatal/perinatal care should receive bereavement and communications training to be able to skillfully assist a parent to make these decisions. This training needs to empower health care workers to respond individually to a range of family needs, ask for feedback in real time, and adjust their communication according to the family’s preferences. Parents recommended drawing on the collective experience, using language such as, “Some families have made this decision for this reason; for other families, they chose a different course because of these important factors.” Timing is challenging. Most participants recommended discussing autopsy, organ donation, and research donation in the context of other decisions about end-of-life care, such as baptism or whether to have family visit. This approach lessens decision fatigue and allows families to think holistically about their parenting choices. In addition, they recommended written materials describing autopsy, organ donation, and research donation. During times of acute stress, it can be difficult to remember details about these procedures. Written materials can also be left with the families to make the decision at their own pace. A word cloud constructed from the coded data of the focus groups highlights the centrality of communication in both the families’ experiences and their recommendations (eFigure in Supplement 1).

**Figure 2.**
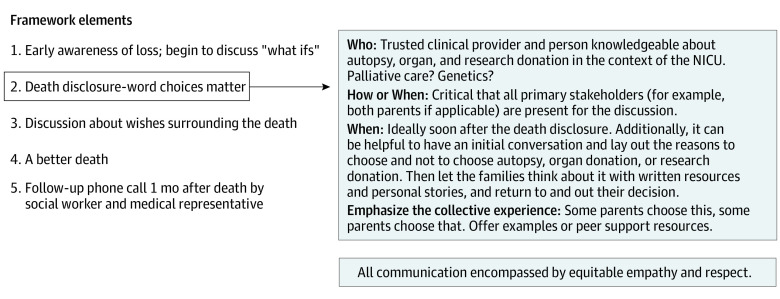
A Recommended Framework for Caring for Families Experiencing Neonatal Loss A recommended framework for caring for families experiencing neonatal loss, according to the findings from this study.

Our findings led to a proposed framework for working with families who experience neonatal loss (Figure 2), encompassing equitable empathy and respect. Focus group participants suggested that clinicians needed to help them become aware earlier that death was possible. One participant felt strongly that this conversation should take place in prenatal appointments, in cases where there is a high likelihood of neonatal death. After a sensitive and thoughtful discussion about their baby’s impending death, clinicians should then help families consider all of their secondary hopes.^24^ In the absence of cure, which is every family’s primary hope, what other desires for their children might we be able to provide in the hospital or as a part of the medical team? With this framing, offering options for autopsy, organ donation, or research donation becomes a natural part of holistic and compassionate end-of-life care. This approach can then facilitate a better adjustment to the baby’s death by the family. One critical aspect to this discussion is that clinicians have local and institutional knowledge about organ and research donation. Clinicians should make every effort to contact the donor network before speaking with families to determine a baby’s eligibility for donation. In some cases, the donor network can also facilitate research.^25^ Finally, engagement with families about autopsy, organ donation, and/or research donation naturally promotes follow-up for families to ensure adequate bereavement care.

## Discussion

In this study, we sought to understand the perspectives of parents who experience neonatal loss on autopsy, organ donation, and research donation. A primary finding in our research is the importance of offering parents the choice of autopsy, organ donation, or research donation with skillful and empathetic communication. Parents in our study endorsed the ability of these options in the setting of neonatal loss to establish a legacy for their deceased child. Furthermore, they offered suggestions to frame autopsy, organ donation, and research donations in ways that support and empower parents rather than further burden or traumatize them. These include providing written materials, emphasizing the collective experience, integrating discussions about the end of life, and having trained and compassionate clinicians who know the family participate in these discussions. A framework for our themes shows how parents’ lived experience can lead to building a legacy, which includes recommendations to integrate posthumous decision-making as a pivotal piece of the medical care for families with neonatal loss (Figures 1 and 2).

Our findings are consistent with prior research^4,26^ that families want to be directed and informed in the processes of neonatal loss. This practice, guided participation, is a cornerstone of individualized, supportive, relationship-based perinatal bereavement care.^27^ Even though these discussions are challenging, they can help promote healing.^10,14,28^ Although not directly investigated in our research, our findings may also help clinicians integrate genetic testing, an increasingly available resource, in the context of neonatal loss.^29,30^

We speculate that discussions about autopsy, organ donation, and research donation help facilitate meaning-making and provide a sense of control, at least over some elements, when a life is lost. Emerging evidence suggests that NICU families and health care workers use different words when describing death.^31^ Future studies should investigate how different phrasing can modify the impact of these discussions on family comprehension and grief processing. Common reasons for avoidance of discussions with parents about autopsy, organ donation, and research donation include lack of training and fear of taking away a family’s hope.^15,16,32^ Our findings add to prior research in perinatal loss, demonstrating that offering autopsy can help provide meaning to the current experience and enable families to feel more informed as they plan for the future.^8,17^ Our study further adds that emphasizing the educational aspects of autopsy is important for some families. It is comforting for them to know that the clinicians will use the autopsy to carefully review the case and potentially change practices in the future. Common reasons cited for parents to decline autopsy include concerns about bodily integrity and dignity, stress of giving permission, objections from other family members, misunderstanding about why autopsy is being requested, religious and cultural objections, and concerns about interference with funeral arrangements.^33,34^ In addition, parents in our study who declined autopsy described a sense that their child’s life was complete without needing the information gained from the autopsy. These factors emphasize the individual nature of appropriate counseling on this topic. Participating in autopsy, organ donation, and research donation is not the right choice for every family. However, informing every family of these possibilities, tailored to the specific options available at each institution, should be a standard part of equitable care for neonatal death.

### Limitations

This study had limitations. We did not capture the perspectives of fathers in this study. Although our sample included racial diversity, the participants were well-educated and had private insurance (Table 1). How preferences for autopsy, organ donation, and research donation may change in individuals from different cultural, educational, or economic backgrounds should be explored in future research.^35,36^ We did not study families whose primary language is not English, but their experiences, feelings, and thoughts about neonatal death and postmortem options are critical to serve patients and their families from diverse cultural backgrounds. In addition, understanding the opinions and experiences of hospital staff regarding autopsy, organ donation, and research donation is an important topic to explore in subsequent studies.

## Conclusions

Participating in autopsy, organ donation, and research donation is not the right choice for every family, but informing every family of these possibilities should be a standard part of equitable care for neonatal death. If the request is made with sensitivity, some families will participate in options that give back to society or the medical system because it is a way to honor their child’s life and build their legacy. Finally, this study highlights the necessity to partner with families to discern their goals and wishes.

## Notes

Supplement 1. **eAppendix.** Focus Groups on Brain Research in the Setting of Neonatal Loss: Facilitator’s Guide **eFigure.** Word Cloud Representing the Codes From Our Focus Group Data and Their Frequency

Supplement 2.
Data Sharing Statement
