# Supplementary material for: Parents’ Views on Autopsy, Organ Donation, and Research Donation After Neonatal Death
Source: JAMA Netw Open. 2023 Nov 6;6(11):e2341533. doi: 10.1001/jamanetworkopen.2023.41533 (PMC10628732; doi:10.1001/jamanetworkopen.2023.41533)
Supplement: Supplement 1. — eAppendix. Focus Groups on Brain Research in the Setting of Neonatal Loss: Facilitator’s Guide eFigure. Word Cloud Representing the Codes From Our Focus Group Data and Their Frequency [file jamanetwopen-e2341533-s001.pdf]

Supplemental Online Content

Crouch EE, Damas C,Bartrug WC. Parents' views on autopsy, organ, and research donation after neonatal death. *JAMA Netw Open*. 2023;6(11):e2341533. doi:10.1001/jamanetworkopen.2023.41533

eAppendix. Focus Groups on Brain Research in the Setting of Neonatal Loss: Facilitator's Guide
eFigure. Word Cloud Representing the Codes From Our Focus Group Data and Their Frequency

This supplemental material has been provided by the authors to give readers additional information about their work.

© 2023 Crouch EE et al. *JAMA Network Open.*

eAppendix. Focus Groups on Brain Research in the Setting of Neonatal Loss: Facilitator's Guide

Session 1

Welcome, and introduction. Introduce Annie as HAND Volunteer coordinator, here to help support participants who may encounter emotions that need processing. I, Dr. Crouch, here to perform research from UCSF.

Remind everyone that I (Dr. Crouch) obtained verbal consent prior to this meeting. Remember that your participation today is completely voluntary and you may stop or skip questions at any time. Do you have any questions about being a research participant? May we begin the recording?

Warm-up activity: participants introduce themselves and share a momento about their child.

Reminder about the purpose of the study: to learn about parents' experiences in being asked about autopsy, organ donation, and research and to provide best practice recommendations for these encounters in the future.

Review the logistics: Today will be the first of two sessions we will have together. In this group today, we'll focus on asking about your experience with autopsy and organ donation, options you may have been asked about or participated in after your baby died. We will spend about 30 minutes talking about each topic. Then, when we come back together in about 4-6 weeks for the second session, we will talk about how doctors and nurses should talk about research with families who have experienced neonatal loss, guided by your thoughts and experiences in this area. We are grateful to be here today to do this work with you. We will not go longer than 90 minutes for each session. Any questions?

Research questions:

I'd like to start by talking about autopsy, which I know can be difficult to think about. Are any of you familiar with autopsy? Can someone tell me what an autopsy is or why it is offered?

From the medical world, which is my experience, autopsy is an exam of the body after death performed by a doctor. Some people choose it because it can provide more information about the life and death of the person. The information could help family members, and it could also help doctors understand diseases better in general.

If you were asked about autopsy, how did the conversation go? What helped you make your decision one way or another? What about the conversation do you wish was different? How do you feel about it now?

© 2023 Crouch EE et al. *JAMA Network Open.*

Very little is known on what NICU family members think about autopsy. Whether or not you wanted or were offered an autopsy, what are your general thoughts on the topic?

Take brief break.

Again, we are so appreciative of your willingness to share your experience. Our second topic is organ donation, which I understand also can be challenging. Are any of you familiar with organ donation? Can someone tell me why it is offered?

From the medical world, organ donation is the process of giving parts of the body after death to help another person. With babies, it can be possible to donate eye lenses and heart valves.

Were any of you able to participate in organ donation? If yes, If you were asked about autopsy, how did the conversation go? What helped you make your decision one way or another? What about the conversation do you wish was different? How do you feel about it now?

Again, very little is known on what NICU family members think about organ donation. Whether or not you wanted or were offered organ donation, what are your general thoughts on the topic?

Closing activity.

Decide on when to meet for the second meeting, if necessary.

Finish with zoom photo, if possible.

Focus groups on brain research in the setting of neonatal loss
Facilitators Guide

Session 2

© 2023 Crouch EE et al. *JAMA Network Open.*

Welcome, and re-introduction. Introduce Annie as HAND Volunteer coordinator, here to help support participants who may encounter emotions that need processing. I, Dr. Crouch, here to perform research from UCSF.

Remind everyone that I (Dr. Crouch) obtained verbal consent prior to this meeting. Remember that your participation today is completely voluntary and you may stop or skip questions at any time. Do you have any questions about being a research participant? May we begin the recording?

Recap from the first meeting.

Review agenda: Today's conversation will focus on helping doctors and scientists ask families who have experienced neonatal loss about research in the most sensitive and thoughtful way. We are as always so grateful that you are choosing to draw from your experience to help us and others in this way. In our first 30 minutes, we will talk about your experiences with research. Then, in the second 30 minutes we will ask about what you think are best ways to bring up this topic with family members who have recently experienced loss.

Research questions:
While some is known about how parents prefer to communicate about autopsy and organ donation, the scientific literature has not really studied how to ask to parents about contributing to research in the setting of loss of a child.

- *First, we want to speak to families with words that honor their child rather than sterilize the experience of their life. When talking about donating tissue, what words should be used? Tissue? Cells? Body?*
- *Was anyone offered the possibility to participate in research after their child passed away? If so, how did the conversation go?*
- *In general, are you in favor or opposed to the idea of including your baby in research after death? Could you please share with us the reasons for your position?*

Brief break.

Who do you think should ask a family if they are interested in research? One of the members of their clinical team (current model in the ICN) or a neutral research coordinator (model in other departments)? Why?

Do you think the person who is asking about research should bring written materials or anything else?

Should the study have a website for more info? Or more directly offer to put families in touch with a scientist (is this allowed via IRB?)

When should parents be asked if they want to participate in research? On admission? When they are eligible for a particular study? Another time?

© 2023 Crouch EE et al. *JAMA Network Open.*

Should the research team have any follow up with the family? If yes, what type of follow-up?

Are there any other questions we should ask or other comments on this topic?

Before we close this session, we would like you to provide feedback on your experience participating in these sessions. What do you think worked well? What do you wish there was more or less of?

Discuss how the results from this work will be summarized and shared with the participants and presented to Community Partners, researchers, and funders. As a reminder, we asked in the consent process if you would be willing to be contacted for future research. If you said “yes”, you may hear from us related to topics that came up from these focus groups.

Closing activity.

© 2023 Crouch EE et al. *JAMA Network Open.*

eFigure. Word Cloud Representing the Codes From Our Focus Group Data and Their Frequency

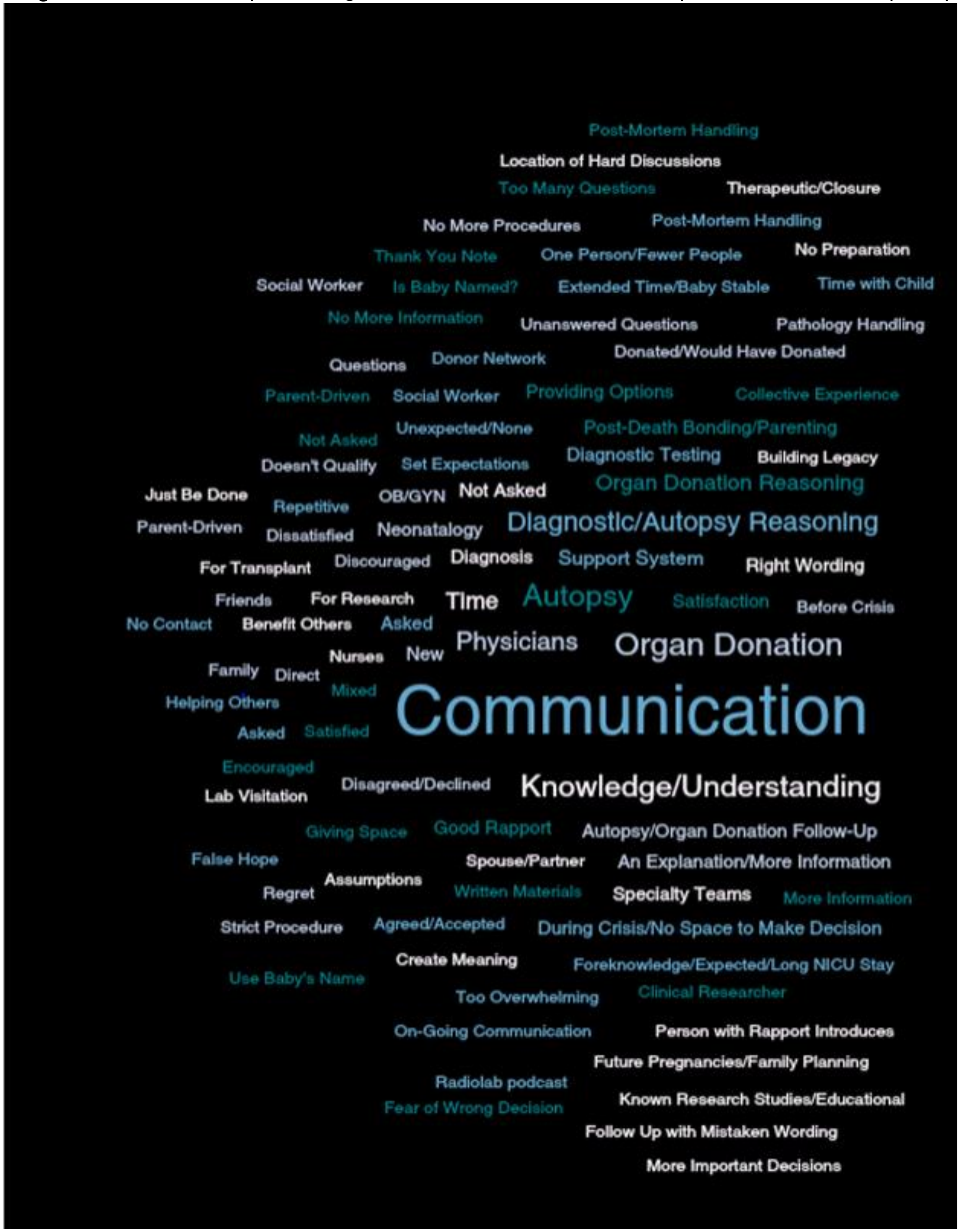

© 2023 Crouch EE et al. *JAMA Network Open.*
