# Supplementary material for: Parents’ Views on Autopsy, Organ Donation, and Research Donation After Neonatal Death
Source: JAMA Netw Open. 2023 Nov 6;6(11):e2341533. doi: 10.1001/jamanetworkopen.2023.41533 (PMC10628732; doi:10.1001/jamanetworkopen.2023.41533)
Supplement: Supplement 2. — Data Sharing Statement [file jamanetwopen-e2341533-s002.pdf]

Data Sharing Statement

Crouch. Parents' Views on Autopsy, Organ, and Research Donation After Neonatal Death. *JAMA Netw Open*. Published November 06, 2023. doi:10.1001/jamanetworkopen.2023.41533

Data
Data available: Yes
Data types: Deidentified participant data
How to access data: All de-identified transcripts are available by emailing the first author, elizabeth.crouch@ucsf.edu.
When available: With publication

Supporting Documents
Document types: Statistical/analytic code
How to access documents: Codes and themes are available by emailing the first author, elizabeth.crouch@ucsf.edu.
When available: With publication

Additional Information
Who can access the data: Requests for data will be discussed by the steering committee, which includes parents who have experienced neonatal loss and medical professionals. All requests for data made in good faith will be honored.
Types of analyses: Coding and thematic analysis.
Mechanisms of data availability: After approval by the steering committee.
